# Cell Therapy in Chagas Disease

**DOI:** 10.1155/2009/484358

**Published:** 2009-06-11

**Authors:** Antonio C. Campos de Carvalho, Regina C. S. Goldenberg, Linda A. Jelicks, Milena B. P. Soares, Ricardo Ribeiro dos Santos, David C. Spray, Herbert B. Tanowitz

**Affiliations:** ^1^Instituto Nacional de Cardiologia, 22240-006 Rio de Janeiro, RJ, Brazil; ^2^Dominick P. Purpura, Department of Neuroscience, Albert Einstein College of Medicine, Bronx, NY 10461, USA; ^3^Instituto de Biofísica Carlos Chagas Filho, Universidade Federal do Rio de Janeiro, 21941-590 Rio de Janeiro, RJ, Brazil; ^4^Department of Physiology & Biophysics, Albert Einstein College of Medicine, Bronx, NY 10461, USA; ^5^Centro de Pesquisas Gonçalo Moniz, Fundação Oswaldo Cruz, 40296-70 Salvador, BA, Brazil; ^6^Department of Pathology, Albert Einstein College of Medicine, Bronx, NY 10461, USA

## Abstract

Chagas disease which is caused by the parasite *Trypanosoma cruzi* is an important cause of cardiomyopathy in Latin America. In later stages chagasic cardiomyopathy is associated with congestive heart failure which is often refractory to medical therapy. In these individuals heart transplantation has been attempted. However, this procedure is fraught with many problems attributable to the surgery and the postsurgical administration of immunosuppressive drugs. Studies in mice suggest that the transplantation of bone-marrow-derived cells ameliorates the inflammation and fibrosis in the heart associated with this infection. Cardiac magnetic resonance imaging reveals that bone marrow transplantation ameliorates the infection induced right ventricular enlargement. On the basis of these animal studies the safety of autologous bone marrow transplantation has been assessed in patients with chagasic end-stage heart disease. The initial results are encouraging and more studies need to be performed.

## 1. Introduction

This year we celebrate the 100th anniversary of the discovery by the Brazilian physician-scientist Carlos Chagas of the disease that bears his name (Chagas disease). This represents a rare instance in the history of medicine where a researcher described the disease, identified the transmission method and isolated the causal agent [1, 2]. Since 1909, many studies have been performed to unravel disease mechanisms and to find a cure for Chagas disease. Unfortunately, although much progress has been achieved, until now there is no consensus on the exact mechanisms that lead to the different manifestations of the disease, nor is there an effective treatment for this infection. 

Chagas disease is caused by the hemoflagellate parasite, *Trypanosoma cruzi*. The parasite has a complex life cycle consisting of different life forms that are distinguished by morphological and biochemical criteria. Blood form trypomastigotes are found in infected mammalian hosts. After the insect vector, from the *Reduvidae* family, ingests blood containing blood form trypomastigotes they transform into dividing epimastigotes in the midgut of the insect vector. After 3–4 weeks epimastigotes become infective, nondividing metacyclic trypomastigotes. These forms are present in the hindgut of the vector and are deposited with the feces during blood meals. Transmission to a new host takes place when the parasite-laden feces contaminate oral or nasal mucous membranes, the conjunctivas, or other vulnerable surfaces such as the skin. In the mammalian host the metacyclic trypomastigotes invade the cells of the host and once inside the parasites transform into intracellular amastigotes that multiply by binary fission. As the amastigotes accumulate inside the host cells, signaling mechanisms that have not been fully identified lead to their transformation into blood form trypomastigotes. They are then released as the host cell ruptures and disseminate through the lymphatics and the bloodstream to invade new cells or, while in circulating blood, may be ingested in meals taken by the insect vectors.

Although any nucleated mammalian cell can be parasitized by *T. cruzi*, cells of the reticuloendothelial, nervous and muscle systems, including the heart, appear to be favored. Chagas disease is characterized by three phases, acute, indeterminate and chronic. In the acute infection, which usually lasts for approximately two months, there are nonspecific signs and symptoms such as fever and myalgias associated with tissue parasitism, inflammation and high peripheral blood parasitemia. The indeterminate phase may last for months to a lifetime during which individuals are relatively asymptomatic. In the chronic phase parasitemia is low or nonexistent but there is an intense inflammatory process in the affected organs. The gastrointestinal tract and heart are the main targets of the chronic stage of the disease and in these organs dilatation is present, constituting the so-called mega syndromes.

The disease is endemic in all Latin America countries with the exception of the Caribbean nations. In these countries it is estimated that 16–18 million individuals are infected with the parasite, with many new cases reported each year. In the past transmission of *T. cruzi * to humans has essentially been vector-borne. Presently this situation has changed drastically due to the successful implementation of vector control programs in many of the endemic countries. The Southern Cone Initiative (SCI), which began in 1991 in Argentina, Bolivia, Brazil, Chile, Paraguay, and Uruguay, was instrumental for the success of the control program. The transmission of *T. cruzi* by blood transfusion has been essentially eliminated throughout much of the endemic range by obligatory testing of donated blood for evidence of *T. cruzi* infection. In recent decades the rate of emigration from Chagas-endemic countries to the United States, Canada, and the European Union has increased markedly. Currently an estimated 100 000 immigrants from these areas are chronically infected with *T. cruzi*. Although these regions are free of the vector transmission, transmission by blood transfusion and by organ transplantation have been reported in Canada and the United States. 

During the chronic phase, cardiomyopathy is the most important clinical manifestation of Chagas disease. It is estimated that 10%–30% of all infected individuals will acquire chronic chagasic cardiomyopathy. This represents anywhere between 1.6 to 5.4 million patients with chronic chagasic cardiomyopathy in Latin America, making Chagas disease one of the most important causes of heart disease in this region. Additionally, the chronic cardiac manifestations of Chagas disease have created an immense social and economic burden in endemic areas because of unemployment and increased health care costs. It has been estimated that 20 000 deaths occur annually in endemic countries due to complications of chronic chagasic cardiomyopathy [3]. 

Dilated congestive cardiomyopathy is an important manifestation of chronic chagasic cardiomyopathy that typically occurs years or even decades after a person first becomes infected. Apical aneurysm of the left ventricle is one of the hallmarks of chronic chagasic cardiomyopathy. Chronic chagasic cardiomyopathy is characterized by focal or disseminated inflammatory infiltrates, myocytolysis, myonecrosis and progressive fibrosis [4, 5]. Remodeling of the myocardium and vasculature is the result of damage to the extracellular matrix and the replacement of cardiac myocytes and/or vascular cells by fibrous tissue. This results in thinning of the myocardium and hypertrophy of the remaining cardiac myocytes and also leads to thromboembolic events. In that regard chronic chagasic cardiomyopathy is similar to other dilated cardiomyopathies that lead to congestive heart failure. The clinical correlation between intensity of the myocarditis varies considerably from mild cardiac symptoms to intense chronic cardiomyopathy, leading to heart failure and death [6]. Patients with chronic chagasic cardiomyopathy may have a variety of arrhythmias causing heart malfunction. The ECG abnormalities include right bundle-branch block, left anterior fascicular block, ventricular premature beats and A-V block [7, 8].

The virtual absence of parasites, both circulating and within the heart and the presence of a focal and widespread inflammatory process in the myocardium have generated multiple hypotheses to explain the etiology of chronic chagasic cardiomyopathy. However, the general opinion is that the etiology of chronic chagasic cardiomyopathy is multifactorial involving parasite persistence, vascular impairment, destruction of ganglia of the autonomic nervous system and autoimmunity [9]. With such complex disease mechanisms it is not surprising that once the cardiomyopathy is established, the prognosis for the chagasic patient is rather bleak. In fact, chronic chagasic cardiomyopathy has been reported to be the main prognostic mortality factor among patients with heart failure of various etiologies [10]. Therapies for chronic chagasic cardiomyopathy are identical to those for congestive heart failure and often include *β*-blockers, diuretics, angiotensin-enzyme inhibitors angiotensin receptor blockers and amiodarone. There is no consensus about the use of anti-trypanosomal agents such as benznidazole. In fact, a large trial designed to address the efficacy of benznidazole in chronic chagasic cardiomyopathy is under way; the BENEFIT Multicenter Trial. As the disease progresses few therapeutic options are left for the chronic chagasic cardiomyopathy patient other than heart transplantation. Although survival in chagasic heart transplant patients has been reported to be longer than that of persons transplanted for heart disease resulting from other etiologies [11], the limited number of donors and the complications of immunosuppressive therapy, including parasite reactivation, make this therapeutic option a very limited one for the majority of chronic chagasic cardiomyopathy patients. In that scenario, cell transplantation appears as an alternative to standard therapies in the setting of chronic chagasic cardiomyopathy.

## 2. Cell Therapy for Chagasic Cardiomyopathy

The use of cell therapy for chagasic cardiomyopathy followed closely the development of research on the use of this therapy in patients with myocardial infarction. The pioneering work of Soonpaa et al. [12] demonstrated conclusively that exogenous cells could be integrated into the host myocardium. Although initially most of the studies in this area of research focused on transplantation of fetal cardiac myocytes, embryonic stem cells, or skeletal myoblasts into hearts that were damaged cryogenically or by myocardial infarction, more recently bone marrow derived cells have become an important cell source. A major development in the use of cell therapies to improve cardiac function was based on the observations that stromal bone marrow cells could be induced to differentiate into cardiac myocytes in vitro [13] and that when they were transplanted into cryo-injured rat hearts, myocardial function improved and angiogenesis was promoted [14]. Another significant development was the report by Orlic et al. [15] that hematopoietic stem cells from transgenic mice expressing enhanced green fluorescent protein (EGFP) transplanted into myocardial infarction-damaged hearts of syngeneic mice differentiated into cardiac muscle and vascular cells. Importantly, they demonstrated complete integration of the transplanted c-Kit^+^ bone marrow derived cells, including formation of connexin 43 gap junctions between the newly formed myocardium and the surviving tissue. Many others have reported that hematopoietic and mesenchymal stem cells derived from bone marrow improve myocardial function in animal models of both cryo-injured and ischemic heart lesions [16–19].

Cardiac regeneration by bone marrow-derived cells has been questioned [20–22] and remains controversial [23]. However, even in cases where cardiac regeneration by bone marrow-derived cells has not been demonstrated, functional measurements have detected improvements in heart function after cell transplantation [22]. More recently, the beneficial effects of cell therapies using bone marrow-derived cells in heart disease have been increasingly attributed to paracrine effects [24, 25].

**Figure 1:**
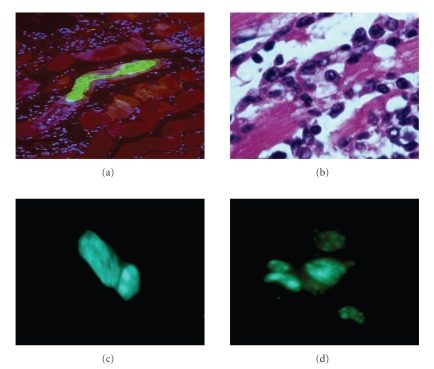
Heart sections of *T. cruzi*-infected mice. (a) BALB/c mouse during the acute phase of infection with Colombian strain *T. cruzi*, showing a parasite nest (green), DAPI-stained nuclei (blue) and myofibers (red). The majority of the DAPI nuclei belong to inflammatory cells that infiltrate the heart in areas infected with parasites. (b) Inflammation of chronic chagasic BALB/c mouse, showing inflammatory cells adhered to myofibers causing myocytolysis. (c) and (d), Detection of bone marrow stem cells (BMC) in the myocardium of chronic chagasic mice. BMC obtained from normal BALB/c mice were injected i.v. into chronic chagasic mice (18 months of infection). BMC were incubated with the fluorescent DNA stain Hoechst 33258 prior to injection into chagasic mice. Sections of frozen heart fragments were prepared 7 (c) and 15 (d) days after BMC injection and fixed with cold acetone. Sections were observed in an Olympus spectral confocal microscope FV1000 observed by fluorescence microscopy. In chagasic mice Hoechst^+^ cells could be observed 1–7 days after BMC injection, some of which were already beginning cell division cycles (c). Hoechst^+^ cells proliferated and formed clusters of cells bearing a dotted nuclear fluorescent pattern that could be observed up to 30 days after BMC transplant (d).

In most of the reported studies the damage to the heart is circumscribed to a specific area since the lesions are ischemic in nature. Accordingly, cell delivery has been mostly intramyocardial, especially in small animals. Due to the global nature of chronic chagasic cardiomyopathy, systemic delivery of cells was chosen for studies in a mouse model of Chagas disease. Our reasoning was that direct myocardial injections would have to be performed in various areas of the left and right ventricle, creating the possibility of myocardial damage due to the multiple injections. Thus, to validate the therapy it was necessary to demonstrate that cells injected intravenously established themselves in the chagasic hearts. In initial experiments bone marrow mononuclear cells were preincubated with Hoechst 33258 stain prior to injection into tail veins of normal and chagasic mice, and cell-treated mice were sacrificed at various time points thereafter. In chagasic mice Hoechst^+^ cells were observed in the heart 1–7 days after BM cell injection, but were not found in heart sections of normal mice injected with Hoechst 33258-stained cells (see Figure 1). Hoechst^+^ cells were also found in the spleen and liver of chagasic and control bone marrow cell-treated mice 1-2 days after transplant. Heart sections of mononuclear cell-treated mice were also stained for stem cell markers by immunofluorescence, and Sca-1^+^ and c-Kit^+^ cell clusters were found in hearts of mononuclear cell-treated mice after cell injection [26]. As a result of these and other experiments in which bone marrow cells from EGFP positive mice were used, it was concluded that bone marrow stem cells home to the chagasic heart, validating systemic injection as a viable approach for cell therapy in this context.

Once the homing of the cells to the diseased myocardium was established, Soares et al. [26] demonstrated that bone marrow mononuclear cells from normal syngeneic donors significantly reduced cardiac inflammation and fibrosis in mice with chronic *T. cruzi* infections. Importantly, the improvement was long lasting, being observed up to six months after cell therapy. The reduction in inflammation likely resulted from increased apoptosis of the infiltrating inflammatory cells as determined by TUNEL staining. The decrease in fibrosis may result from activation of metalloproteases, since MMP9 expression is increased in the chagasic hearts after cell therapy. Cell dosing experiments demonstrated that 10^5^ cells were necessary for a significant reduction in the number of inflammatory cells and injection of 10^6 ^ or 10^7^ cells induced similar effects [26].

The mechanisms of action of the mononuclear cells in chagasic mouse hearts have not yet been fully elucidated. Trans differentiation/fusion appears to occur at an extremely low frequency and paracrine effects may be the major cause of improvement in myocardial function. In clinical human trials autologous bone marrow cells would be employed. Therefore, bone marrow cells from chronically infected mice were used to ameliorate the pathology of infected mice [26]. These experiments highlight the translational aspects of these animal studies.

 Recently, using cardiac MRI, it was demonstrated that tail vein injection of 10^7^ bone marrow mononuclear cells prevented and reversed the right ventricular dilatation induced by *T. cruzi*-infection [27] which correlates with the pathologic improvement reported by Soares et al. [26]. Furthermore it was determined that repeated injections of Granulocyte-colony stimulating factor (G-CSF), which mobilizes stem cells from the bone marrow, decrease inflammation and fibrosis in the hearts of chagasic mice Garcia et al. personal communication. This finding is consistent with observations of Harada et al. [28] that demonstrated improvement in heart function in an ischemic mouse model. The combination of mononuclear cells and G-CSF enhances the effect of the cell therapy in the reduction of the inflammatory infiltrate.

In a rat model of chagasic cardiomyopathy Guarita et al. [29] reported that direct left ventricular injection of cocultured skeletal myoblasts and mesenchymal bone marrow derived cells improved heart function in chronically infected rats as determined by echocardiography. Injection of the cocultured cells increased ejection fraction and decreased end-systolic and diastolic volumes. These findings demonstrated that local injection of stem cells is also effective and suggest that cells are able to diffuse from the injection site to reach other regions of the heart. This is an important observation, given the widespread involvement of the myocardium in chagasic cardiomyopathy. 

Based on the encouraging results in animal models, investigators in Brazil initiated a clinical trial to examine the feasibility and safety of autologous bone marrow cell transplantation in patients with congestive heart failure due to chronic chagasic cardiomyopathy. These patients generally have a poor prognosis, with mortality rates reaching 40% within two years of onset [30]. At the most advanced stage of congestive heart failure the only therapy possible is heart transplantation, but this procedure is feasible in only a very small number of patients. Due to uncertainties regarding the mechanisms of action of the mononuclear cells, the trial was designed for patients with end-stage congestive heart failure whose only therapeutic option would be heart transplantation. This was an open label, uncontrolled, single center clinical trial that enrolled 30 patients. Inclusion criteria required patients to be 18–70 years old, of either gender, with congestive heart failure due to Chagas' disease, in New York Heart Association (NYHA) class III or IV, with an ejection fraction of less than 40% while on optimized pharmacologic therapy for at least 4 weeks before enrollment [31]. Bone marrow cell aspiration was performed on the day of the injection and the mononuclear fraction was obtained through Ficoll density gradient centrifugation. The cell suspension was diluted in 20 mL of saline with 5% autologous serum and injected in the coronary arteries using an angioplasty catheter with the following distribution: 10 mL in the left descending coronary artery, 5 mL in the circumflex and 5 mL in the right coronary artery. Mean number of injected cells was 2.7 × 10^8^. At the 25th day after cell injection patients received 5 *μ*g/kg of G-CSF for 5 days. Patients were followed for six months. Importantly there was no detectable increase in arrhythmias after cell therapy nor were troponin I levels increased during or after the procedure. Results indicated that cell therapy induced a small but significant increase in ejection fraction. Quality of life improved as determined by the Minnesota Questionnaire and by NYHA class. The six minute walking test also showed significant improvement. These results were observed 1 month after therapy and persisted for the 6 month follow-up period. However, since the trial was not designed to test for efficacy the only conclusion possible is that bone marrow mononuclear cell therapy by intracoronary delivery is feasible and safe in chronic chagasic cardiomyopathy patients.

In a patient with chagasic cardiomyopathy, bone marrow mononuclear cells delivered by the intracoronary route were preferentially retained in diseased, hypoperfused areas of the myocardium [32]. Further studies using labeled cells confirmed these results (Barbosa, personal communication).

Given the promising results of the phase II trial, a larger, multicenter, randomized, double-blind and placebo controlled trial was designed to test for efficacy of the intracoronary delivery of bone marrow-derived mononuclear cells in chronic chagasic cardiomyopathy. Inclusion criteria included diagnosis of heart failure by the Framingham criteria, regular visits to a cardiology service with at least two independent serological diagnoses of Chagas disease, ages between 18–75 years, NYHA class III or IV, ejection fraction below 35% by echocardiography according to Simpson's rule, and optimized pharmacologic therapy. Main exclusion criteria were valvular diseases (except for functional mitral or tricuspid regurgitation), coronary angiography with significant lesions (more than 50% of obstruction), sustained ventricular tachycardia, abusive use of drugs or alcohol, serum creatinine >2.5 mg/dl, neoplasia and other diseases that might impact life expectancy within two years. Primary endpoint for the trial is the difference in ejection fraction between the cell therapy and the placebo group as determined by Simpson's rule in echocardiography. The trial was powered to detect an absolute 5% difference as significant. Secondary endpoints include difference in ejection fraction, life quality assessment by Minnesota Quality of Life Questionnaire, six minute walking distance, NYHA class and brain natriuretic peptide levels at baseline and 6 and 12 months after therapy, among others. The trial is still recruiting patients, but initial 6 month follow-up results are expected to be published by July 2009.

Transplantation of bone marrow derived-cells may prove to be an important therapeutic modality in the management of end-stage chagasic heart disease. Undoubtedly, identifying which cell type(s) is(are) responsible for the effects observed in the animal and the preliminary human experiments will be an important step toward improvement of this therapy. Since the percentage of stem cells, either hematopoietic or mesenchymal, is minimal in the mononuclear fraction, use of purified stem cell populations has the potential to significantly increase the therapeutic potential of cell therapy in chagasic cardiomyopathy.
